# How do manufacturing and producer service agglomerations affect urban innovation differently? Empirical evidence from China

**DOI:** 10.1371/journal.pone.0275616

**Published:** 2022-10-12

**Authors:** Mingdou Zhang, Weilu Li, Rui Zhang, Xia Yang

**Affiliations:** 1 School of Geographical Sciences, China West Normal University, Nanchong, China; 2 School of Economics, Dongbei University of Finance and Economics, Dalian, China; Instituto Tecnologico Autonomo de Mexico, MEXICO

## Abstract

Despite the growing body of literature on the influence of industrial agglomeration on urban innovation, no consensus has been reached on the mechanism of the spillover effect. This empirical study exploits heterogeneity in spillover effects between manufacturing and producer service agglomerations on urban innovation based on a sample of 262 prefecture-level cities in China. We find some intriguing and new findings: (1) The threshold effect can be identified for the spillover effect of manufacturing agglomeration but not for that of producer service agglomeration. (2) Manufacturing and producer service agglomerations have opposite decomposition indirect effect. (3) The spatial spillover effect of industrial agglomeration can be restrained by absorptive capacity of nearby cities. This study not only provides empirical evidence for the reconciliation of the debate on the effect of manufacturing and producer service agglomeration, but also has important policy implication for reconsidering the role of industrial agglomeration in urban innovation.

## 1. Introduction

With the deterioration of the international market environment and the shackles of domestic production factor endowments, China’s economy has entered a new normal development stage, and high-quality development has become a new era feature of China’s economic growth. Technological innovation plays an important role in realizing the transformation of China’s economy from scale and speed to quality and efficiency. Relevant research shows that innovation is the establishment of a new production function, which introduces a new combination of production factors and production conditions into the production system [1]. In this way, innovation can reduce the production cost of enterprises, improve the market competitiveness of enterprise products, help develop new products and new technologies, optimize and upgrade the industrial structure, meet the changing demand structure, and promote the continuous development of the economy [2]. As a space carrier for national innovation activities, cities gather various innovation elements and innovation resources, providing an important place for innovation output [3]; Therefore, urban innovation has received extensive attention from academia, and research results are more focused on the analysis of the influencing factors of urban innovation, of which industrial agglomeration is an important factor [4–6]. Industrial agglomeration makes a large number of enterprises concentrated in the same geographical space, and enterprises as the main body of innovation, face-to-face and various innovation resources exchange and penetration, dynamic cumulative reinforcement learning is more likely to induce breakthrough innovation [7]. The reason is that geographically adjacent innovation subjects often face similar policy environments and cultural backgrounds. It is easier for different subjects to communicate with each other to promote the spillover of knowledge and technology, and the flow of innovation elements is also smoother, which makes innovation in this region easier. The positive interaction between subjects and organizations forms a formal or informal relationship network, and strengthens the collaborative and efficient operation between innovation subjects and organizations, thereby accelerating the research and development and diffusion of new technologies and new knowledge, and improving the overall level of innovation in the region [8,9].

Research on the relationship between industrial agglomeration and innovation has a long history. The technological externalities caused by industrial agglomeration are divided into two categories. One is the specialized economy represented by Marshall, Arrow, and Romer, which advocates that the internal agglomeration of the same industry is conducive to the maximum possession and application of innovation achievements; the other is the diversified economy represented by Jacobs, which believes that the agglomeration of different industries is conducive to the formation of an innovation environment with complementary and different characteristics. Subsequent scholars mostly conduct empirical analysis on the impact of specialized agglomeration and diversified agglomeration on innovation. Due to differences in research objects and empirical methods, research conclusions are not the same. One school of scholars supports that specialized economy can effectively increase the frequency of innovation activities [10–12]. Another school of scholars supports a diversified economy that facilitates the collision and exchange of knowledge and technologies with different characteristics, and is more likely to stimulate innovation [13–15]. However, studies on the impact of industrial agglomeration on urban innovation from the perspective of manufacturing and producer services are still insufficient. Although the only studies have confirmed that manufacturing agglomeration and producer service industry agglomeration can provide diversified labor and intermediate inputs and other innovative elements, promote competition and exchanges and cooperation between different enterprises, and thus stimulate and promote regional innovation [16]. However, due to the limited market capacity and environmental carrying capacity, and the scarcity of innovative resources such as labor force and infrastructure construction, there is an upper limit on the scale of industrial agglomeration. If the scale of industrial agglomeration exceeds the optimal scale, a crowding effect will occur, which is not conducive to the improvement of regional innovation capabilities [17]. Therefore, there may be a complex nonlinear relationship between industrial agglomeration and the level of urban innovation [18]. Not only that, this paper also believes that there is a spatial effect of industrial agglomeration, because knowledge and technology are both subordinate to the main body of innovation, and the main body of innovation itself has the characteristics of flow, which leads to the phenomenon of knowledge spillover and technology diffusion [19]. Therefore, the positive externalities induced by industrial agglomeration will affect the innovation behavior of neighboring cities, and the degree of influence is regulated by the difference in absorptive capacity between cities. At present, manufacturing agglomeration and producer service industry agglomeration have increasingly become the focus of international industrial competition in the global production network and the focus of global industrial layout adjustment. In-depth exploration of the relationship between manufacturing agglomeration, producer service industry agglomeration and urban innovation level has very important practical significance and research value.

The contributions of this paper to the existing literature are mainly from three aspects as follows. First, as both positive and negative spillover effects of industrial agglomeration on urban innovation have been found in previous studies, we propose and justify the reconciliation by means of the threshold effect. Second, unlike most existing studies, we examine the spatial spillover effects of the influence of manufacturing and producer service agglomerations on urban innovation with absorptive capacity taken into consideration as an influencing factor. Third, we explore the heterogeneity of the impact of manufacturing and producer services agglomeration on urban innovation for different regional spaces in views of scale and level of development in industrial agglomeration.

The rest of the paper is organized as follows. Section 2 provides the literature review and research hypothesis. Section 3 describes variable selection and model specification. Section 4 presents empirical results with discussions. Section 5 concludes the paper.

## 2. Literature review and research hypothesis

### 2.1. Manufacturing agglomeration and local urban innovation

The manufacturing industry is mainly engaged in product processing and manufacturing industry, and its geographic spatial agglomeration is mainly to promote technological innovation and progress to improve the level of urban innovation. The influence of manufacturing agglomeration on technological innovation is due to the geographical proximity effect of innovation main body. To be specific, geographical space adjacent to reduce the risk of innovation main body is engaged in the activities, also facilitate the innovation main body between information and knowledge, especially tacit knowledge transfer and spread, so as to expand the cluster innovation network and promote the innovation output [20]. The promoting effect of manufacturing agglomeration on technological innovation has reached a consensus in the academic circles, but there are differences about the mechanism of manufacturing agglomeration promoting technological innovation. Marshall [21] emphasized that the geographical concentration of enterprises within the same industry facilitated the exchange and dissemination of knowledge, thus the improvement of industrial expertise accelerated knowledge spillover and stimulated innovation activities. On the contrary, Jacobs [22] believes that knowledge spillover mainly occurs between different industries, and industrial diversification agglomeration in geographical space is more conducive to promoting technological innovation. There have been a lot of researches on these two mechanisms in academic circles. One view is that Marshall externalities are good for innovation. Gerben and Cees [23] found that the specialization externality of industrial agglomeration is obvious, while the diversification externality is not. Li et al. [24] found that specialized agglomeration can promote the dissemination of creative knowledge, promote technical cooperation between enterprises, and also help to reduce transaction costs and improve transaction efficiency, which will undoubtedly have a positive impact on urban innovation. Another view is that Jacobs externalities are conducive to innovation. The research results of Glaeser, et al. [25] show that important knowledge spillover may occur between industries rather than within them. Andersson, et al. [26] believe that diversity is crucial to innovation, especially in the manufacturing industry. Similarly, taking Italian manufacturing firms as the research object. Antonietti and Cainelli [27] find that diverse agglomeration can significantly promote regional innovation. There is also a view that Marshall externalities and Jacobs externalities are both beneficial to innovation. Pacil and Usai [28] used the European regional innovation activity database to study the impact of specialization externalities and diversification externalities on innovation output. The results show that the two externalities are not opposed to each other, but jointly stimulate innovation activities of enterprises within the region.

As a labor-intensive and capital-intensive industry, the agglomeration of manufacturing industry in geographical space will inevitably bring about the rapid concentration of labor, capital and other elements in urban space. However, due to the insufficient supply of public infrastructure and public services, a large number of labor forces and the rapidly increasing demand for resources and energy exceed the existing capacity of the city. At this time, the agglomeration economy brought by industrial agglomeration began to turn to the crowding effect, and the negative externality to technological innovation began to appear. Facts have proved that once the scale of industrial agglomeration exceeds a certain threshold, the crowding effect will exceed the effect of agglomeration and have a negative impact [29]. At present, the negative externality of industrial agglomeration has also attracted the attention of many scholars. The research results of Audretscht and Feldman [30] show that with the expansion of industrial agglomeration, enterprises in the same industry tend to adopt a vicious competition for the development of other enterprises in geographical proximity in order to seize the market and deliberately hinder the exchange and cooperation of knowledge and technology, which is not conducive to technological innovation. Brakman et al. [31] proved that the "crowding effect" brought by excessive industrial agglomeration would change the equilibrium distribution of economic activities and produce external diseconomy in the agglomeration area by constructing the general equilibrium model. In addition, Rizov et al. [32] believe that in areas with the high level of urbanization and the density of economic activities, urban resource elements are relatively insufficient, and the cost of knowledge innovation and technological research and development of enterprises is high, which reduces the enthusiasm of enterprises for innovation and research and development, it is not conducive to the improvement of urban innovation level in the long run. In conclusion, this paper argues that manufacturing agglomeration scale plays a moderating role in the relationship between manufacturing agglomeration and urban innovation level. When the scale of manufacturing agglomeration is small and has not reached the optimal agglomeration scale, industrial agglomeration will promote technological innovation and improve the level of urban innovation. With the expansion of agglomeration scale, once the optimal agglomeration scale is exceeded, the "crowding effect" brought by manufacturing agglomeration will inhibit technological innovation and weaken the level of urban innovation. In view of this, this paper makes the following assumptions:

*Hypothesis 1*: *There is a threshold effect on the influence of manufacturing agglomeration on urban innovation level*.

### 2.2. Producer service agglomeration and local urban innovation

The Producer services are industries that provide services for manufacturing enterprises. The agglomeration of producer services in geographical space can promote the improvement of urban innovation level through two ways. First, the agglomeration of producer services can directly promote the improvement of urban innovation level. Keeble & Nacham [33] argued that since producer services are knowledge-intensive industries, their spatial agglomeration can explore agglomeration benefits from the perspectives of agglomeration learning and knowledge spillover. With the agglomeration of producer services, a large amount of knowledge, technology and information gather in space. The mutual contact and communication between innovation subjects can accelerate the diffusion of knowledge and technology, form effective complementarity of knowledge, and finally improve the innovation ability of enterprises through knowledge spillover. And, in the form stable exchange and the cooperation between enterprises, on the basis of gradually set up technical cooperation and exchange platform, enterprises can be shared by advantage resources, technical advice, technical services and other research and development facilities, improve the utilization rate of resources, reduce the innovation cost, improve the efficiency of innovation, which makes the area to enjoy as a result of external economies of scale.

Second, it indirectly influences the level of urban innovation by promoting technological innovation in manufacturing industry. It has been proved that the agglomeration of producer services promotes the technological innovation of manufacturing industry through the effect of scale economy and technology spillover, but the scale economy and technology spillover formed by different forms of industrial agglomeration show different connotations. According to scholars represented by Marshall [21], the agglomeration of specialization in the same industry promotes technological innovation in manufacturing industry through scale economy effect and technology spillover effect. On the one hand, the intermediate service market formed by the specialized agglomeration of producer services can provide specialized producer services for manufacturers. The proximity of different enterprises in geographical location promotes the face-to-face communication between producer service enterprises and manufacturing enterprises, saves transportation costs and transaction costs, and forms economies of scale. On the other hand, with the specialization of producer services agglomeration, a large number of master similar productive service techniques of employees in the same area quickly, make between producer services and manufacturing in the formal or informal learning communication more convenient, promote information and technology sharing between, is advantageous to the manufacturing enterprise technological innovation, improve production efficiency and competitiveness. The scholars represented by Jacobs [22] believed that the diversified agglomeration of different industries could also generate economies of scale and technology spillover to promote the improvement of technological innovation level in manufacturing industry. Firstly, diversified agglomeration of producer services can provide diversified intermediate inputs to meet the needs of the development of the manufacturing industry, so that the manufacturing industry can realize economies of scale in the sharing of intermediate services [34]. Secondly, diversified agglomeration of producer services promotes mutual learning and communication among heterogeneous enterprises, which is conducive to the innovation of existing knowledge and technology, the accumulation and diffusion of complementary and differentiated knowledge, and ultimately leads to the improvement of enterprises’ innovation ability.

The agglomeration of producer services can directly and indirectly promote the improvement of urban innovation level. However, some scholars believe that the positive influence of producer services agglomeration on urban innovation level is significant in the early stage of agglomeration, but with the continuous expansion of producer services agglomeration scale, such positive incentive will gradually disappear and even turn into a inhibiting effect, which is in line with Williamson hypothesis [35,36]. However, considering that the development of producer services in China is relatively lagging behind, the scale of agglomeration needs to be further expanded. Moreover, producer services, as technology-intensive and knowledge-intensive industries with high added value, have a higher capacity for producer services compared with capital and labor-intensive industries, that is, the threshold value is higher. To sum up, this paper makes the following assumptions:

*Hypothesis 2: Producer service agglomeration has a positive spillover effect on local urban innovation, but no threshold effect is expected*.

### 2.3. Industrial agglomeration and urban innovation of neighboring cities

For a long time, knowledge spillover and technology diffusion have been considered to play an important role in improving the level of regional innovation [30,37]. When innovation resources such as knowledge and technology accumulated by a city cannot be fully utilized by itself, the interregional flow of innovation resources will appear. In other words, knowledge and technology generated in one city can be spread to other cities through spatial spillover effect and become a source of local innovation, rather than just due to its own efforts [38]. This is consistent with the conclusion found by Acs, et al. [39] that innovation activities in one region may be affected by neighboring regions, thus leading to spatial correlation. Because knowledge transmission and technology diffusion must be carried out through direct, unintentional and repeated human contact [40,41]. Therefore, the spatial spillover of knowledge and technology is bound to be limited by geographical factors. Not only that, the spillover effect may be influenced by the combination of other factors. For example, Gonçalves and Almeida [42] believe that there is a relationship between regional knowledge spillover and urban hierarchy, and continuous urban hierarchy is conducive to the diffusion of knowledge spillover. Zheng et al. [43] found that infrastructure and policy factors also contribute to spillover effects. Ning, et al. [7] pointed out that foreign direct investment is an important source for developing countries to acquire external knowledge, and its spatial spillover effect depends on the intensity of industrial agglomeration within and between the invested cities. In addition, there are some scholars believed that the absorptive capacity of the members in the cluster directly affects the spillover effect of the aggregated knowledge, that is, the absorptive capacity acts as a mediator on the knowledge spillover [44,45]. Specifically, absorptive capacity affects the absorption cost of knowledge and technology, and the stronger the absorptive capacity of the recipient of spatial spillover is, the more conducive it is to knowledge spillover and technology diffusion. However, due to the different mechanisms of manufacturing agglomeration and producer services agglomeration on the level of urban innovation, the spatial spillover of knowledge and technology between neighboring cities may have different effects on the externality of manufacturing agglomeration and producer services agglomeration. In view of this, this paper makes the following assumptions:

*Hypothesis 3: Industrial agglomeration has spatial spillover effect on the urban innovation of neighboring cities, and the effect depends on absorption capacity and differs between manufacturing and producer service agglomerations*.

## 3. Variable description and model specification

### 3.1. Variable description

We adopt averaged number of patent grants as the proxy of urban innovation, and use local entropy to measure industrial agglomeration. The selection of control variables is in line with the literature. The detailed variable description is as follows.

### Dependent variable

The level of urban innovation (denoted as *inv*). Several measures have been used to quantify the level of urban innovation, among which the average number of patent grants is most intuitive and conventionally used [46]. We take the number of patent grants per 10,000 people as the measure of urban innovation.

### Independent variable

Industrial agglomeration. Location entropy is used to measure manufacturing (denoted as *agg_manu*) and producer service agglomerations (denoted as *agg_serv*). Specifically, it is the ratio of the number of employees in the manufacturing or producer service industry to the number of local employees in each city divided by the proportion of the number of employees in the manufacturing or producer service industry in the total number of employees in the country, which can be expressed as follows:

$$agg_{ij}=\frac{x_{ij}/\sum_{i}x_{ij}}{\sum_{j}x_{ij}/\sum_{i}\sum_{j}x_{ij}},$$ (1)

where *agg*_*ij*_ is the agglomeration degree in industry *i* of city *j*, *x*_*ij*_ is the number of employments in industry *i* of city *j*. To alleviate the controversy about the definition of the producer service industry, we select the industries of transportation, storage, post, and telecommunications; information transmission computer service and software; financial; leasing and business service; and scientific research, technical service, and geological survey to represent the producer service industry.

### Control variables

Government behavior (denoted as *gover*), urban economic development level (denoted as *eco*), opening to the outside world (denoted as *open*), fixed asset investment level (denoted as *fix_cap*), human capital level (denoted as *labor*), and information level (denoted as *inform*). Foreign direct investment is adjusted by exchange rates to alleviate the fluctuation in exchange market. Table 1 shows the summary of the variables.

**Table 1:** Variable description.

	Variable	Symbol	Variable description
Dependent variable	City innovation level	*inv*	Number of patent authorizations owned by 10,000 people
Independent variables	Manufacturing concentration	*agg_manu*	Number of manufacturing employees in each city as a percentage of local employees / manufacturing employees as a percentage of national employees
	Agglomeration of producer services	*agg_serv*	Percentage of people engaged in producer services in each city as a percentage of local employees/Proportion of people engaged in producer services in national employees
Control variables	Government Action	*gover*	The proportion of fiscal expenditure to GDP
	The level of economic development	*eco*	GDP per capita
	Opening to the outside world	*open*	The proportion of foreign direct investment in GDP
	Fixed asset investment level	*fix_cap*	The proportion of the whole society’s fixed capital investment in GDP
	Urban human capital level	*labor*	Number of students in ordinary colleges and universities
	Information level	*inform*	The proportion of total post and telecommunications business in GDP

### 3.2. Spatial matrix construction and autocorrelation test

(1) Spatial matrix construction. With the fast development of Internet technology, cities are becoming closely interrelated. Thus, restricting research objects to only geographically neighboring cities seems inappropriate. We use the latitude and longitude to calculate the spherical distance between two cities to construct the spatial weight matrix *W* as follows:

$$W_{ij}=W_{ji}=\{\begin{array}{l} \frac{1}{d^{2}},\;i\neq j\\ 0,\;i=j \end{array},$$ (2)

where *W*_*ij*_ and *W*_*ji*_ also represent the distance weight between cities *i* and cities *j*, *d* represents the spherical distance between two cities.

Considering that the difference in absorptive capacity between the two cities has influence on the spatial spillover effect, we also create the economic distance spatial weight matrix W* = WΨ. Ψ denotes the difference in absorption capacity of neighboring cities as follows [47].

$$\Psi_{ij}=\{\begin{array}{l} \frac{1}{\left|adop_{i}-adop_{j}\right|},\;i\neq j\\ 0,\;i=j \end{array},$$ (3)

where Ψ_*ij*_ represents the difference in absorptive capacity between city *i* and city *j*, *adop*_*i*_ and *adop*_*j*_ represent the average absorption capacity of cities *i* and *j* in 2008–2017 and are measured by government technology expenditure. The smaller the gap between the absorptive capacities of the two cities, the greater the weight value.

(2) Spatial autocorrelation test. Spatial measurement methods can only be used if spatial autocorrelation exists. To examine the spatial dependence between data, we follow existing literature to calculate the Moran’s I index [48,49], which is defined as follows:

$$Moran’s\;I=\frac{\sum_{i=1}^{n}\sum_{j=1}^{n}W_{ij}(x_{i}-\bar{x})(x_{j}-\bar{x})}{S^{2}\sum_{i=1}^{n}\sum_{j=1}^{n}W_{ij}},$$ (4)

Where *x*_*i*_ represents the innovation level of city *i*, *x*_*j*_ represents the innovation level of city *j*, $\overset{¯}{x}$ represents the mean value of the innovation level of all cities, $S^{2}=\frac{1}{n}\sum_{i=1}^{n}{\left(x_{i}-\bar{x}\right)}^{2}$. The value range of the index is [−1,1] with positive (negative) value indicating positive (negative) correlation. An index equal to 0 indicates that the spatial distribution is random and no spatial correlation exists.

### 3.3. Spatial model specification

In order to test whether the spatial effect is caused by spatial lag correlation or spatial residual correlation, most of the existing domestic studies use a combination of Moran index, LM_Lag, RLM_Lag (spatial lag robustness test), LM_Error and RLM_Error (spatial error robustness test) to select the model. The results of LM test are shown in Table 2: all the null assumptions are rejected based on the linear model without spatial effect (except LM_Lag). Therefore, both the SAR model and the SEM model can be accepted, in which case SDM (spatial Durbin model) is usually given priority [50].

**Table 2:** Spatial econometric model testing.

Model	*Moran’s I*	*LM_Error*	*RLM_Error*	*LM_Lag*	*RLM_Lag*	*LR_SAR*	*LR_SEM*	*Wald_SAR*	*Wald_SEM*
Spatial model	309.968***	9.122***	16.152***	1.775	8.805***	49.21***	145.49***	48.94***	116.45***

Note

***, **, and * indicate significance at 1%, 5%, and 10% levels, respectively.

Wald test and LR test are used to verify whether SDM models can be degenerated into SAR models or SEM models. As shown in Table 2, it is found that both Wald value and LR value reject the null hypothesis. Therefore, SDM model can well describe the spatial correlation of urban economic efficiency. Based on the Hausman test, the fixed effect model was adopted. Following Rocchetta & Mina[51], we take log-transformation to all variables and include the one-period lagged independent and control variables to alleviate the potential endogeneity problem and reflect the time lag effect of the independent variables. Explicitly, the models are shown in (5) and (6):

$$\begin{array}{l} inv_{it}=\beta_{0}+\rho inv_{it}+\beta_{1}agg\_manu_{t-1}+\beta_{2}agg\_serv_{t-1}+\beta_{3}gover\\ \;+\beta_{4}eco+\beta_{5}open+\beta_{6}fix\_cap+\beta_{7}labor+\beta_{8}inform\\ \;+\beta_{9}W\times agg\_manu_{t-1}+\beta_{10}W\times agg\_serv_{t-1}+u_{i}+\varepsilon_{it}, \end{array}$$ (5)

$$\begin{array}{l} inv_{it}=\beta_{0}+\rho inv_{it}+\beta_{1}agg\_manu_{t-1}+\beta_{2}agg\_serv_{t-1}+\beta_{3}gover+\beta_{4}eco+\beta_{5}open\\ \;+\beta_{6}fix\_cap+\beta_{7}labor+\beta_{8}inform+\beta_{9}sqagg\_manu_{t-1}+\beta_{10}sqagg\_serv_{t-1}\\ \;+\beta_{11}W\times agg\_manu_{t-1}+\beta_{12}W\times agg\_serv_{t-1}+\beta_{13}W\times sqagg\_manu_{t-1}\\ \;+\beta_{14}W\times sqagg\_serv_{t-1}+u_{i}+\varepsilon_{it}, \end{array}$$ (6)

where *ε*_*it*_ = *N*(0,*δ*^2^), *inv* is the level of urban innovation, *agg_manu* is the degree of manufacturing agglomeration, *agg_serv* is the the degree of agglomeration of producer services, *gover* is government action, *eco* is the level of economic development, open is the level of openness, *fix_cap* is the level of investment in fixed assets, *labor* is the level of human capital, *inform* is the level of information, and *W* is the spatial weight matrix.

## 4. Empirical results and analysis

### 4.1. Descriptive statistics

A sample of 262 prefecture-level cities in China covering the period of 2008–2017 is used for the empirical study. The data are taken from *China Statistical Yearbook* at the provincial level, *China City*, and *China Regional Economic Statistical Yearbook* from 2008 to 2018. Some indicators are manually compiled from the statistical yearbook year by year. The geometric growth method is applied for handling missing values. Table 3 shows the descriptive statistics with 2,620 observations for each variable.

**Table 3:** Descriptive statistics.

Variable	Mean	Standard deviation	Minimum	Maximum
*inv*	1.495	1.324	-1.609	4.377
*agg_manu*	-0.385	0.844	-2.411	1.470
*agg_serv*	-0.262	0.654	-1.447	1.551
*gover*	2.762	0.397	1.905	3.829
*eco*	10.505	0.604	9.103	11.863
*open*	2.031	1.916	0.010	13.810
*fix_cap*	4.232	0.376	3.075	4.989
*labor*	4.598	1.109	1.532	7.057
*inform*	0.754	0.491	-0.620	2.175

### 4.2. Spatial autocorrelation test results

As shown in Table 4, the Moran’s I values of urban innovation are positive from 2008 to 2017 and significant at 1% level. This finding implies obvious spatial positive correlation, that is, cities with similar innovation levels have significant spatial agglomeration effects [52].

**Table 4:** Moran’s I test.

Year	2008	2009	2010	2011	2012	2013	2014	2015	2016	2017
Moran’s I	0.362*** (14.21)	0.372*** (14.92)	0.428*** (16.82)	0.437*** (17.66)	0.430*** (17.29)	0.482*** (18.96)	0.501*** (19.44)	0.478*** (18.55)	0.473*** (18.30)	0.509*** (19.77)

*Note*s: The numbers in parentheses are *z* values

***, **, and * indicate significance at 1%, 5%, and 10% levels, respectively.

**Fig 1:**
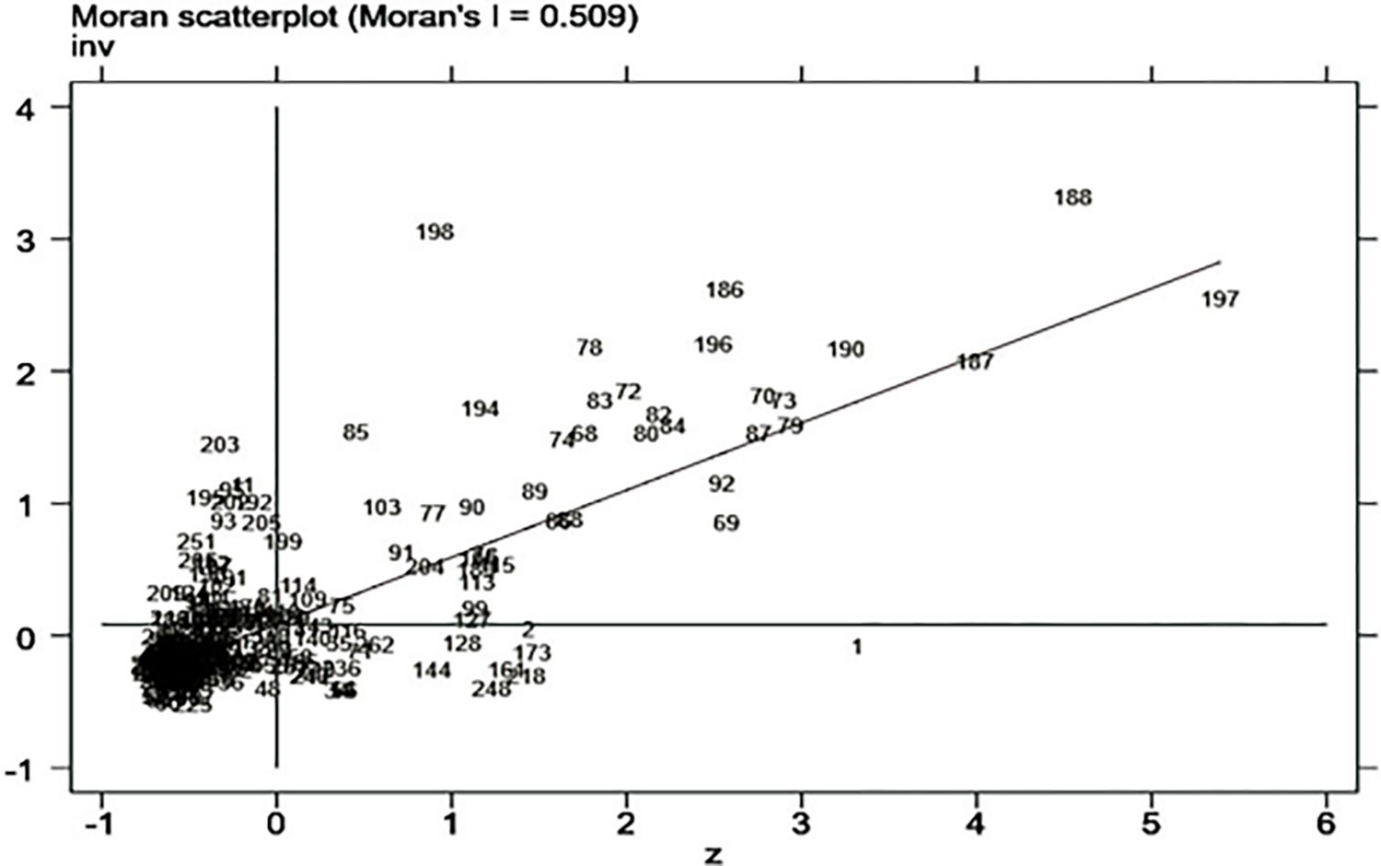
Moran scatterplot of Chinese cities’ innovation level in 2017.

We use Moran scatterplot to further examine the geographical distribution of urban innovation. Fig 1 shows the Moran scatterplot for urban innovation in 2017. Each number in the figure represents the level of innovation in different cities. The first quadrant is H-H agglomeration, which means that cities with higher innovation levels are surrounded by cities with higher innovation levels; the second quadrant is L-H agglomeration, which means that cities with lower innovation levels are surrounded by cities with higher innovation levels; the third quadrant is L-L agglomeration, which means that cities with a low level of innovation are surrounded by cities with a low level of innovation; the fourth quadrant is H-L agglomeration, which means that cities with a high level of innovation are surrounded by cities with a low level of innovation. The local Moran index of the first and third quadrants is positive, indicating that there is a positive spatial correlation; the local Moran index of the second and fourth quadrants is negative, indicating that there is a negative spatial correlation. Obviously, the innovation level of most cities falls in the first and third quadrants, that is, there is a positive spatial correlation between the innovation levels of different cities, which is consistent with the positive number of the global Moran index.

### 4.3. Spillover effect of industrial agglomeration on local urban innovation

Table 5 shows the results of the spatial Durbin model based on the geographic and the economic distance spatial weight matrices. We have two main observations. First, manufacturing agglomeration has a significantly positive spillover effect on urban innovation. The above results imply that China’s manufacturing agglomeration is in an ascending stage and has not yet reached the optimal agglomeration scale. In other words, the agglomeration of manufacturing industry is still promoting urban innovation. The current spatial agglomeration of China’s manufacturing industry is obvious, but it is in an imbalanced situation. Most of the agglomerations are concentrated in the southeast coast, and the overall spillover effect has not been fully exerted. Similarly, the effect of producer services agglomeration on urban innovation level in China is mainly positive incentives. At present, China’s producer services show a good trend of accelerating development, the types of services are constantly improved, the structure is further optimized, and the agglomeration scale continues to increase. However, as a whole, China’s producer service industry is not at a high level of development and lacks international competitiveness, so it still has great potential and space for development.

**Table 5:** Spatial Durbin model regression results.

	Geographic distance spatial weight matrix			Economic distance spatial weight matrix		
	Estimated coefficient	direct effect	indirect effect	Estimated coefficient	direct effect	indirect effect
*ρ*	0.632*** (21.68)			0.429*** (17.13)		
*agg_manu*_*t-*1_	0.075*** (3.26)	0.099*** (4.09)	0.756*** (4.93)	0.085*** (3.68)	0.107*** (4.39)	0.377*** (5.38)
*agg_serv*_*t-*1_	0.157*** (4.39)	0.129*** (3.45)	-0.825*** (-2.97)	0.167*** (4.57)	0.151*** (4.01)	-0.239* (-1.89)
*gover*	0.144** (2.25)	0.145** (2.40)	-0.176 (-0.68)	0.046 (0.73)	0.085 (1.46)	0.574*** (4.23)
*eco*	0.636*** (8.44)	0.681*** (9.36)	1.414*** (9.04)	0.592*** (7.86)	0.654*** (9.19)	1.063*** (10.24)
*open*	-0.006 (-0.59)	-0.010 (-0.92)	-0.102* (-1.65)	-0.008 (-0.74)	-0.013 (-1.21)	-0.082** (-2.57)
*fix_cap*	-0.031 (-0.73)	-0.028 (-0.68)	0.051 (0.30)	-0.088** (-2.08)	-0.071* (-1.78)	0.253** (2.76)
*labor*	0.065** (2.04)	0.069** (1.99)	0.119 (0.58)	0.064* (1.94)	0.060* (1.72)	-0.062 (-0.58)
*inform*	0.031 (1.45)	0.041** (1.97)	0.336** (2.22)	0.036* (1.66)	0.040* (1.91)	0.091 (1.13)
$W\times agg\_manu_{t-1}$	0.234*** (4.22)			0.187*** (4.44)		
$W\times agg\_serv_{t-1}$	-0.410*** (-4.08)			-0.215*** (-2.86)		
*Individual effect*	Π			Π		
*R* ^*2*^	0.702			0.624		
*Log_likelihood*	-558.174			-809.008		
*Number of samples*	2620			2620		

*Note*s: The numbers in parentheses are *z* values

***, **, and * indicate significance at 1%, 5%, and 10% levels, respectively.

### 4.4. The threshold effect of industrial agglomeration

As debated in existing literature, industrial agglomeration can have positive and negative effects on urban innovation. On the one hand, industrial agglomeration can improve the level of urban innovation through the spillovers of knowledge and technology. On the other hand, excessive industrial agglomeration can produce the crowding effect, leading to excessive competition, increased production costs, reduced production efficiency, and inhibited urban innovation. To examine whether the effect of industrial agglomeration on urban innovation has an inverted-U pattern, we include the square terms of the agglomeration degree of the manufacturing and the producer service industries in the model.

Table 6 shows the results of the threshold effect examination based on the geographic and the economic distance spatial weight matrices. The threshold effect can be clearly observed for the spillover effect of manufacturing agglomeration on local urban innovation, indicating the gradual change from promoting effect to crowding effect with the increase in agglomeration level. Meanwhile, no threshold effect can be identified for the spillover effect of producer service agglomeration on local urban innovation.

**Table 6:** Threshold effects of industrial agglomeration on local urban innovation.

	Geographic distance spatial weight matrix			Economic distance spatial weight matrix		
	Estimated coefficient	direct effect	indirect effect	Estimated coefficient	direct effect	indirect effect
*ρ*	0.623*** (21.19)			0.428*** (17.14)		
*agg_manu*_*t-*1_	0.022 (0.84)	0.038 (1.36)	0.485*** (3.15)	0.025 (0.95)	0.041 (1.44)	0.263*** (3.45)
*sqagg_manu*_*t-*1_	-0.050*** (-3.83)	-0.058*** (-4.12)	-0.280*** (-2.85)	-0.057*** (-4.20)	-0.061*** (-4.27)	-0.092** (-2.17)
*agg_serv*_*t-*1_	0.166*** (4.59)	0.142*** (3.81)	-0.687** (-2.38)	0.176*** (4.78)	0.166*** (4.42)	-0.129 (-0.91)
*sqagg_serv*_*t-*1_	0.019 (0.71)	0.037 (1.26)	0.573** (2.36)	0.031 (1.11)	0.048 (1.62)	0.306*** (2.73)
*gover*	0.161** (2.52)	0.167*** (2.82)	-0.007 (-0.02)	0.065 (1.06)	0.106* (1.86)	0.609*** (4.14)
*eco*	0.657*** (8.71)	0.697*** (9.53)	1.216*** (7.65)	0.613*** (8.15)	0.670*** (9.41)	0.990*** (9.53)
*open*	-0.007 (-0.63)	-0.009 (-0.87)	-0.071 (-1.14)	-0.008 (-0.74)	-0.012 (-1.22)	-0.064** (-1.96)
*fix_cap*	-0.039 (-0.93)	-0.040 (-0.97)	-0.087 (-0.53)	-0.099** (-2.35)	-0.085** (-2.09)	0.207** (2.30)
*labor*	0.076** (2.39)	0.084** (2.41)	0.244 (1.17)	0.077** (2.36)	0.076** (2.15)	-0.020 (-0.18)
*inform*	0.030 (1.40)	0.038* (1.81)	0.279* (1.88)	0.034 (1.59)	0.038* (1.81)	0.088 (1.08)
$W\times agg\_manu_{t-1}$	0.176*** (2.94)			0.149*** (3.28)		
$W\times sqagg\_manu_{t-1}$	-0.079** (-2.21)			-0.032 (-1.26)		
$W\times agg\_serv_{t-1}$	-0.377*** (-3.73)			-0.158** (-1.99)		
$W\times sqagg\_serv_{t-1}$	0.212** (2.37)			0.172*** (2.70)		
*Individual effect*	Π			Π		
*R* ^*2*^	0.697			0.713		
*Log_likelihood*	-546.866			-591.084		
*Number of samples*	2620			2620		

*Note*s: The numbers in parentheses are *z* values

***, **, and * indicate significance at 1%, 5%, and 10% levels, respectively.

### 4.5. Spillover effect of industrial agglomeration on neighboring urban innovation

Columns 2 to 4 in Table 5 show the results based on the spatial weight matrix regression of geographic distance. Combined with indirect effects, we can find that manufacturing agglomeration has a significantly positive spatial spillover effect on urban innovation level, whereas producer service agglomeration has exactly the opposite spatial spillover effect on urban innovation.

As an industry that provides necessities for other industries to upgrade and undergo technological progress and improve production efficiency, the producer service’s agglomeration level accelerates the agglomeration of other industries. Consequently, some manufacturing industries are likely to be located in areas with comprehensive producer services. The agglomeration of producer service industries attracts the industries of neighboring cities, and hence has negative impact on the development of neighboring cities. This situation is undoubtedly unbeneficial to the improvement of the innovation level of neighboring cities and is particularly typical in the central region of China. For example, as the national central city and the core city of the middle reaches of the Yangtze River, Wuhan has a strong secondary industry foundation and a high level of producer service industry concentration. As neighboring cities, Xianning, Xiaogan, and Huanggang are likely to have benefitted from Wuhan’s more advanced producer services to develop their own industries. However, the development of these cities is not as good as Shiyan and Xiangyang, which are cities far away from Wuhan. The concentration of producer services attracts other industries in surrounding cities to settle in Wuhan. This phenomenon, so called “black under the lights”, is by far rare, with Changsha and Taiyuan served as typical examples.

### 4.6. Influence of absorptive capacity on spatial spillover effect

As shown in Table 5, the spatial autoregressive coefficients *ρ* under both spatial weight matrices are significant at 1% level. This finding indicates a significant spatial spillover effect of industrial agglomeration on urban innovation.

Considering the difference in absorption capacity between cities on the spatial spillover effect of industrial agglomeration, we construct the economic distance spatial weight matrix (W*) for further analysis. As shown in Table 5, absorption capacity has a significant effect on the spatial spillover effect of industrial agglomeration, with significant drops in the spatial autoregressive coefficient. This finding indicates that the greater the gap between the absorptive capacities of two regions, the more conducive they are to the spillover of knowledge and technology and the positive spillover of the level of innovation. There is empirical evidence that absorption capacity is a factor for the improvement in innovation level of the technology absorption side; however, stronger absorption capacity for the technology spillover side means stronger imitation ability, which may lead to intensified competitions and potentially huge losses [53]. Therefore, narrowing the gap in absorptive capacity motivates protection of technology, but it is not beneficial for the spillover of knowledge and technology. In addition, due to the influence of differences in absorptive capacity, the regression coefficients of the indirect effects of manufacturing agglomeration and producer service agglomeration all show a decrease in varying degrees, which further proves that absorptive capacity has a significant impact on the spatial spillover effect of industrial agglomeration.

### 4.7. Robustness check

To estimate the reliability of the results, which is in view of the manufacturing industry agglomeration and the effectiveness of producer services agglomeration how to affect the level of city innovation. This paper will conduct robustness test from the following three aspects, and the regression results are shown in Tables 7 and 8. Firstly, considering that in China’s urban governance hierarchy structure, the political and economic resources tend to municipalities directly under the central government and provincial capital cities, and with other cities as there are differences in the degree of industrial concentration and innovation level. Therefore, the samples of provincial capitals and municipalities were removed from Model 1 for estimation. The results show that: from the direct effect, the regression coefficients of manufacturing agglomeration and producer services agglomeration are significantly positive; from the indirect effect, the regression result of manufacturing agglomeration is significantly positive, while that of producer services agglomeration is significantly negative. Considering the difference of absorptive capacity of neighboring cities, the spatial autoregressive coefficient ρ decreases, which confirms the robustness of the main conclusions in this paper. Secondly, considering the impact of city size on urban innovation, the expansion of city size will stimulate the market demand and strengthen the degree of financial agglomeration, and then stimulate the improvement of urban innovation level. Therefore, Model 2 takes the total population at the end of the year as the proxy variable of city size and adds it to the regression model. The results show that the basic conclusion of this paper remains unchanged. Finally, this paper divides the national sample into two groups, the eastern region and central and western regions, and regression results are shown in Model 3 and Model 4. The eastern region includes Beijing, Tianjin, Hebei, Liaoning, Shanghai, Jiangsu, Zhejiang, Fujian, Shandong, Guangdong and Hainan, and the central and western regions include Shanxi, Inner Mongolia, Jilin, Heilongjiang, Anhui, Jiangxi, Henan, Hubei, Hunan, Sichuan, Guizhou, Yunnan, Chongqing, Shaanxi, Gansu, Qinghai, Ningxia, Xinjiang, Guangxi. The above approach can not only serve as a robustness test, but also analyze whether the agglomeration of manufacturing and producer services in different regions of China has different effects on the level of urban innovation.

**Table 7:** Robustness check based on geographic weight matrix.

	Model 1		Model 2		Model 3		Model 4	
	direct effect	indirect effect	direct effect	indirect effect	direct effect	indirect effect	direct effect	indirect effect
*ρ*	0.648*** (21.58)		0.577*** (18.41)		0.422*** (8.93)		0.618*** (19.37)	
*agg_manu*_*t-1*_	0.090*** (3.47)	0.781*** (4.56)	0.082*** (3.50)	0.539*** (3.98)	0.041 (1.04)	0.192* (1.69)	0.139*** (4.47)	0.856*** (4.82)
*agg_serv*_*t-1*_	0.151*** (3.64)	-0.882*** (-2.88)	0.103*** (2.86)	-0.949*** (-3.94)	0.155** (2.16)	-0.529* (-1.84)	0.147*** (3.24)	-0.261 (-0.98)
*gover*	0.127** (1.97)	-0.301 (-1.09)	0.114** (1.90)	-0.420* (-1.72)	0.341*** (3.06)	0.147 (0.57)	0.088 (1.21)	0.565* (1.87)
*eco*	0.659*** (8.56)	1.480*** (8.60)	0.636*** (8.55)	1.225*** (8.77)	1.358*** (11.63)	0.328 (1.56)	0.376*** (4.06)	1.442*** (8.67)
*open*	-0.013 (-1.18)	-0.098 (-1.48)	-0.007 (-0.72)	0.001 (0.02)	-0.002 (-0.11)	-0.223 (-0.78)	-0.019 (-1.51)	-0.062 (-0.84)
*fix_cap*	-0.080* (-1.80)	0.159 (0.89)	-0.039 (-0.96)	-0.143 (-1.02)	-0.055 (-0.85)	-0.023 (-0.14)	0.020 (0.38)	0.019 (0.10)
*labor*	0.061 (1.58)	0.131 (0.55)	0.079** (2.31)	0.487*** (2.58)	-0.034 (-0.49)	-0.242 (-0.99)	0.080** (1.97)	0.191 (0.93)
*inform*	0.040* (1.76)	0.353** (2.10)	0.046** (2.23)	0.387*** (2.92)	-0.003 (-0.08)	0.192 (1.39)	0.035 (1.40)	-0.064 (-0.37)
*pop*			-0.190 (-1.27)	5.818*** (7.24)				
*R* ^*2*^	0.700		0.390		0.637		0.581	
*Log_likelihood*	-565.189		-529.660		-130.794		-391.420	
*Individual effect*	Π		Π		Π		Π	
*Number of samples*	2330		2620		990		1630	

*Note*s: The numbers in parentheses are *z* values

***, **, and * indicate significance at 1%, 5%, and 10% levels, respectively.

**Table 8:** Robustness check based on economic distance weight matrix.

	Model 1		Model 2		Model 3		Model 4	
	direct effect	indirect effect	direct effect	indirect effect	direct effect	indirect effect	direct effect	indirect effect
*ρ*	0.356*** (12.42)		0.414*** (16.21)		0.275*** (8.01)		0.513*** (16.95)	
*agg_manu*_*t-1*_	0.103*** (3.88)	0.338*** (4.94)	0.102*** (4.26)	0.342*** (4.90)	0.052 (1.35)	0.079 (1.04)	0.133*** (4.28)	0.506*** (4.68)
*agg_serv*_*t-1*_	0.171*** (4.07)	-0.409*** (-3.18)	0.145*** (3.92)	-0.288** (-2.35)	0.164** (2.29)	0.186 (1.03)	0.161*** (3.59)	-0.093 (-0.57)
*gover*	0.073 (1.12)	0.379*** (2.73)	0.082 (1.41)	0.506*** (3.60)	0.328*** (2.97)	0.191*** (4.61)	0.045 (0.64)	0.535*** (2.68)
*eco*	0.731*** (9.88)	1.081*** (10.46)	0.666*** (9.14)	0.987*** (9.86)	1.016*** (9.00)	0.503*** (8.46)	0.310*** (3.53)	1.450*** (11.68)
*open*	-0.018 (-1.60)	-0.074** (-2.52)	-0.010 (-0.93)	-0.068** (-2.05)	-0.022 (-1.05)	-0.011 (-1.31)	-0.023* (-1.82)	-0.030 (-0.62)
*fix_cap*	-0.086* (-1.92)	0.205** (2.27)	-0.076* (-1.91)	0.200** (2.29)	-0.138** (-2.34)	-0.058** (-2.55)	-0.009 (-0.17)	0.190 (1.49)
*labor*	0.057 (1.45)	0.004 (0.04)	0.078** (2.21)	0.026 (0.24)	-0.034 (-0.49)	-0.011 (-0.36)	0.055 (1.37)	-0.042 (-0.32)
*inform*	0.039* (1.70)	0.108 (1.44)	0.043** (2.02)	0.098 (1.23)	0.001 (0.02)	0.002 (0.09)	0.029 (1.16)	-0.071 (-0.68)
*pop*			0.342** (2.35)	1.383*** (2.72)				
*R* ^*2*^	0.667		0.682		0.631		0.624	
*Log_likelihood*	-665.787		-595.960		-124.318		-403.262	
*Individual effect*	Π		Π		Π		Π	
*Number of samples*	2330		2620		990		1630	

*Note*s: The numbers in parentheses are *z* values

***, **, and * indicate significance at 1%, 5%, and 10% levels, respectively.

According to the empirical results, the manufacturing agglomeration in the central and western regions has a significant promoting effect on the innovation level of cities, and manufacturing agglomeration has a significant role in promoting the innovation level of neighboring cities in the eastern and central and western regions. In addition, the influence coefficient of the agglomeration of producer service industry in the eastern region and central and western regions on the innovation level of cities is significantly positive, and the regression coefficient of the agglomeration of producer service industry in the eastern region on the innovation level of adjacent cities is significantly negative. The absorptive capacity has a significant influence on the spatial spillover effect of industrial agglomeration, and the spatial auto regression coefficient ρ has decreased to different degrees in the eastern region and central and western regions. These conclusions prove the robustness of the empirical results of the spatial Durbin model in Table 5. It is worth noting that the manufacturing agglomeration in the eastern region no longer has a significant promoting effect on the urban innovation level. The regression coefficient of producer services agglomeration on the innovation level of neighboring cities is negative in the central and western regions, but not significant. The main reasons are as follows.

Since the reform and opening up, the spatial pattern of China’s manufacturing industry has undergone changes under combined influences of marketization and globalization. The southeast coast has been developed as the main region for industrial agglomeration [54]. At the same time, the eastern coastal areas attract a large number of foreign-funded enterprises due to their geographical and institutional advantages, which further strengthens the industrial agglomeration level within the region, thus laying the basic pattern of highly industrial agglomeration in the coastal areas. In this spatial pattern, the agglomeration level of manufacturing industry in eastern China is constantly improving and gradually approaching the threshold value. Some cities even have the phenomenon of production factors crowding, which means that the efficiency of industrial agglomeration is not economic. Therefore, it weakens the promoting effect of manufacturing agglomeration on urban innovation level. On the whole, producer services in China have developed to a new height, and their contribution rate to economic growth exceeds that of the manufacturing industry. However, there is still a big gap in the development level of producer services in different regions. Producer services in southeast coastal areas have a relatively high development level, while the central and western regions are still in the stage of vigorously developing manufacturing, so the agglomeration level of producer services is relatively low, and it cannot produce "siphon" effect on industries in neighboring cities.

## 5. Conclusion

This empirical study uses a sample consisting of 262 prefecture-level cities in China covering the period of 2008–2017 to analyze the influence of manufacturing and producer service agglomerations on urban innovation and examine the threshold and the spatial spillover effects. To compare and analyze the regional differences in the influence of manufacturing and producer service agglomerations on the level of urban innovation, the national sample is further divided into the eastern, central and western regions.

Our main conclusions are as follows. (1) The influence of manufacturing agglomeration on urban innovation level presents an obvious inverted-U shape, confirming the existence of the threshold effect; The agglomeration of producer services has a significant promoting effect on the level of urban innovation, but the threshold effect has not yet emerged. Manufacturing agglomeration has a positive effect on the innovation level of neighboring cities, but producer services agglomeration has a significant inhibiting effect on the innovation level of neighboring cities. (2) The influence of industrial agglomeration on the level of urban innovation has a significant spatial spillover effect. The difference in the absorption capacities of adjacent cities weakens this spatial spillover effect. (3) The regression coefficient of manufacturing agglomeration on urban innovation level in eastern China is positive but not significant, but it has a significant promoting effect on the innovation level of neighboring cities. Manufacturing agglomeration in central and western China has a significant promoting effect on the innovation level of cities, and also has a significant promoting effect on the innovation level of neighboring cities. In eastern China, producer services agglomeration has a significantly positive impact on urban innovation level, while the regression coefficient on the innovation level of neighboring cities is significantly negative. In central and western China, producer services agglomeration has a significantly positive impact on urban innovation level, while the regression coefficient on the innovation level of neighboring cities is negative but not significant.

Our study also provides several policy implications. (1) *Overdone is worse than undone*. For urban development, moderate industrial agglomeration can contribute to urban innovation. If the agglomeration exceeds the optimal level, the threshold effect occurs, which brings in adverse effect on urban innovation. Therefore, full consideration to the urban resource endowments is significant when making development strategies of industrial agglomeration. In this way, an appropriate level of agglomeration can be maintained without reaching excessive industrial concentration. (2) *Win-win is better than lose-lose*. The result of the vicious competition between neighboring cities is the consequence of lack in synergy and efficiency for the urban development system, which requires strengthened cooperation between cities. Through cooperation and exchanges between cities, the boundaries of administrative divisions can be broken, and the formation of urban agglomerations can be promoted. As a mutually beneficial developmental pattern, integration drives the formation of the entire urbanized area. (3) *Suit measures to local conditions*. The problem of uneven regional development in China requires localized policy-making for urban development. The eastern coastal area has attracted a large number of enterprises because of its location and institutional advantages. Its industrial agglomeration level is also relatively high. Therefore, producer service agglomeration in this region is suitable to create a long-term service base for the development of other industries and the improvement in production efficiency. By contrast, the central and western regions are still at the stage of accelerating urban agglomeration economy. These regions should enhance their agglomeration economic effects by promoting agglomeration of manufacturing industry, which in turn stimulate urban innovation.

The limitations of the paper are mainly reflected in two aspects. First, spatial weight matrix is the basis of spatial statistical analysis, which has gradually attracted the attention of geographers. In this paper, an assumption is introduced when constructing the spatial weight matrix: *W*_*ij*_ = *W*_*ji*_. However, the reality is different. Suppose there exist two cities *i* and *j*. The economic and social development level of city *i* is far higher than that of city *j*, and the development of city *j* greatly depends on city *i*, while the development of city *i* has a low dependence on city *j*. In this case, *W*_*ij*_ ≠*W*_*ji*_, and the assumption is not valid. Obviously, the spatial weight matrix constructed based on the hypothesis cannot well describe the interdependence between the two cities. How to solve the above problems has been discussed in the academic circles to a certain extent, but unfortunately no consensus has been formed. In the future, relevant research should combine some typical characteristics of respective cities, comprehensively consider the realistic situation and research operability, and construct a more scientific and realistic spatial weight matrix. Second, the scope of producer services is not precise enough. Producer services include transportation, modern logistics, financial services, information services, high-tech services, business services, etc. However, producer services cannot be fully defined due to the absence or serious absence of some industry data in the statistical yearbook. How to define the scope of producer services more comprehensively and accurately and scientifically quantify it becomes an urgent problem to be solved in future research.
